# The evolutionary features and internal logic of the one-vote veto system: A Nvivo analysis based on the policy texts of the three provinces in the east, middle and west

**DOI:** 10.1371/journal.pone.0306535

**Published:** 2024-07-05

**Authors:** Jiancheng Li

**Affiliations:** School of Humanities and Social Science, University of Science and Technology Beijing, Beijing, China; CNR: Consiglio Nazionale delle Ricerche, ITALY

## Abstract

Analyzing the evolutionary features and internal logic of the one-vote veto system in China over the past two decades is highly significant when considering reform and standardization. In order to conduct this analysis, the Nvivo 12 software was used to examine policy texts related to the one-vote veto issued by Fujian, Hubei, and Gansu provinces. Through a comparative analysis of keyword frequency statistics, policy text form, and content characteristics across the three provinces, it was discovered that governmental departments have experienced fundamental changes in their utilization of the one-vote veto system after 20 years of development. These changes are primarily seen in the refinement of the description of the one-vote veto in policy texts, the gradual reduction in the withdrawal mechanism of the one-vote veto, and an expanded application field for the one-vote veto.

## 1. Introduction

In early March 2019, the General Office of the Central Committee of the Communist Party of China issued a notice addressing the important issue of reducing formalism and alleviating the burden on grassroots organizations. This notice, referred to hereafter as “the Notice”, declared 2019 as the year dedicated to reducing burdens at the grassroots level. It emphasized that *governments at all levels must strictly control matters that require one-vote veto*, *refrain from signing responsibility certificates arbitrarily*, *and avoid disguising their responsibility by transferring it to local and grassroots entities* [1]. The one-vote veto system serves as a crucial component of the performance evaluation system unique to China. Its purpose is to encourage grassroots governments to effectively implement key policies mandated by the central government, utilizing a mechanism with strong disincentives. Its aim is to curtail the autonomy of lower-level governments and incentivize prompt action toward fulfilling the directives of higher-level authorities. Within the macro-institutional framework of the bureaucratic government system, the one-vote veto system relies on personnel management authority and is interdependent with the target responsibility system. By associating the outcomes of performance appraisals with political recognition, economic rewards, and career advancements for grassroots governments and their officials, this unconventional yet rigid incentive measure establishes a severe system of accountability. In essence, the essence of the one-vote veto system is that if a grassroots government fails to meet the requirements set by higher-level authorities in executing a specific task, the higher-level government will completely disregard any achievements made in other areas. Consequently, officials from the relevant departments and their superiors may face severe penalties such as suspension, demotion, or dismissal. The one-vote veto system can be likened to a constant threat hanging over the heads of grassroots government officials, akin to a sharp sword.

If counting from 1982 when Changde City, Hunan Province took the lead in implementing the one-vote veto system in family planning, this system has been implemented in China for more than 40 years. Its scope of use has also gradually expanded from the initial four items of economic development, family planning, social security, energy conservation and emission reduction to education and scientific research, urban construction, tax collection and many other fields. As a means of assessment and accountability with Chinese characteristics, the one-vote veto system did play a certain role in overcoming the short-sighted cognitive bias and administrative delay in local governments at the beginning of its implementation. However, by generalizing the one-vote veto indicator, the negative effects of this reverse incentive tool gradually emerged, causing alienation in the behavior of local government officials, leading to the prevalence of no-accident logic and responsibility-avoiding behavior in grassroots governance [2, 3]. The current one-vote veto system, resembling an administrative firefighting approach, no longer meets the requirements of contemporary administrative management models. Hence, there is an urgent need for the revision and innovation of this system. In light of this, this study employs the text analysis method using Nvivo software to examine the contents of pertinent policy documents related to the one-vote veto system issued by Fujian, Hubei, and Gansu provinces between 2000 and 2019. Through a differentiated analysis of the policy expressions employed by these three provinces, this paper aims to summarize the evolution characteristics and internal logic of China’s one-vote veto system over the past twenty years. Ultimately, this research seeks to offer a feasible theoretical reference for innovating and standardizing this system.

## 2. Literature review

The one-vote veto system has a longstanding history of over 40 years in China, and its application field continues to expand. However, despite the widespread enthusiasm for this institutional tool in practical contexts, academic discussions on the topic remain limited. A search through various literature retrieval systems reveals that by the end of 2022, there were less than 400 authoritative sources available in both Chinese and foreign languages pertaining to the one-vote veto. Existing research primarily focuses on describing the system’s application status in specific fields and discussing its function and nature.

### 2.1 Research on the problem of one-vote veto in specific fields

Academic research on the issue of one-vote veto in specific fields is closely related to the application of this system in practical fields. The one-vote veto system was initially applied in the field of family planning. Driven by this system, China’s family planning undertaking has made brilliant achievements [4]. An empirical study by Gu et al. based on data from 420 prefecture-level administrative units in China showed that China’s average fertility rate had dropped to 1.47 at the end of the 20th century, which was far below the population replacement level [5]. The success of the one-vote veto for family planning has accelerated the spread of the one-vote veto system across the country, and has been applied in areas such as environmental protection, energy conservation and emission reduction. Based on the political promotion observation data of 810 municipal leading cadres from 2005 to 2015, Tang et al. revealed the threshold effect of environmental pollution on official economic competition from the perspective of the one-vote veto system [6]. A study by Wu et al. based on pollutant emission data from 286 cities also shows that through the one-vote veto system, the Chinese government links energy conservation and emission reduction targets by promoting local officials. This significantly changes the decision-making trade-off between developing the economy and protecting the environment among local government officials [7]. Since then, in order to correct the downward shift in the bottom line of teachers’ ethics among teachers, the one-vote veto system has been applied to the construction of ethics. Li et al. used content analysis to study China’s teachers’ ethics construction policy and found that over time, the regulations on the use of the one-vote veto system in China’s teachers’ ethics construction have become increasingly clear [8]. This will help expand and deepen the value of the school’s teacher ethics construction policy [9]. In recent years, with the country’s increasing emphasis on rural issues, the application of the one-vote veto system in targeted poverty alleviation work has become more common and has received widespread attention from scholars. Based on an in-depth study of four typical counties for poverty alleviation and development in Guizhou Province, Wang found that integrating the one-vote veto system as a guarantee into the basic requirements for improving the autonomy of grassroots organizations in poverty alleviation work is of typical significance for poverty alleviation and development in concentrated contiguous extremely poor areas [10]. Obviously, in view of the simplicity and remarkable effect of the one-vote veto system, this system has been widely used in social governance in many fields in the course of more than forty years since its birth. Such an abusive situation has led scholars to cry out that *the one-vote veto is like a basket*, *and everything can be put in it* [11], which in turn triggered discussions in academic circles about the fundamental function and nature of the one-vote veto system.

### 2.2 Research on the function and nature of the one-vote veto system

An important reason why the research on the one-vote veto system has not attracted enough attention from scholars is that the current academic circle has not yet formed a relatively unified understanding of the function and nature of the system, and there are completely different views on its role in the process of grassroots governance. One point of view is that the one-vote veto system has played an important role in advancing the central government’s important and difficult tasks and in promoting China’s economic and social development [12]. Fu et al. examined the impact of China’s professional incentive model on pollutant emissions based on panel data of provincial leaders and found that the use of a one-vote veto system in environmental pollution assessment significantly led to changes in the incentive structure [13]. This means that, as a mandatory assessment system for local governments by the central government, the system can effectively overcome the short-sighted cognitive bias of local governments when making inter-temporal choices. To a certain extent, adjusting the local chief executive’s policies and initiatives to promote urban development [14]. However, another view is that the one-vote veto system lacks a sufficient and complete basis at the philosophical level [15]. Moreover, as a cadre selection and appointment mechanism, it has not formed a significant binding force in practice [16]. A study by Wang et al. based on panel data on local government environmental governance in the Yangtze River Delta region found that although the financial pressure caused by the system significantly improved the efficiency of local government environmental governance, it also weakened the behavioral preferences of local governments to develop the local economy [17]. Obviously, the abuse of this system weakens the real central work of grassroots governance [18]. Therefore, such a pre-modern administrative model that does not conform to scientific management principles should be abandoned. In addition, some scholars have pointed out, based on the investigation of China’s river chief system and the practice of grassroots government public service provision, that since the one-vote veto system has played a certain role in China’s performance management assessment, it cannot be abandoned lightly [19]. However, it is necessary to know the appropriateness of the one-vote veto, and strictly regulate its use scenarios with a reasonable and effective institutional framework design [20].

### 2.3 Review of existing literature

Scholars have conducted extensive and thorough research on the one-vote veto system, and these research findings have provided a solid theoretical foundation for this paper. However, there are still some shortcomings in the existing research. Firstly, the academic studies on the one-vote veto problem mainly focus on discussing its attributes, neglecting a systematic review of the related policy texts. Secondly, due to the limitation of the research object, previous literature relies heavily on normative research methods, lacking a comprehensive analysis of the evolution characteristics and internal logic of the one-vote veto system using textual quantitative analysis. To address these deficiencies, this paper aims to improve existing research in two aspects. Firstly, it will summarize and analyze the policy texts related to the one-vote veto issued by provinces in eastern, central, and western China from 2000 to 2019. By comparing the similarities and differences between these texts, the paper will identify the evolution characteristics and internal logic of China’s one-vote veto system. Secondly, in terms of research methods, this paper will employ the text analysis method based on Nvivo software to conduct standardized quantitative analysis of relevant policy texts. This will involve encoding keywords, counting high-frequency words, creating word cloud maps, building semantic networks, among other techniques.

## 3. Research methods and data processing

### 3.1 Research methods

This study primarily utilizes the Nvivo software for text analysis in order to investigate pertinent issues. Text analysis is a content analysis method that combines qualitative and quantitative methods. It is a general term for a class of methods that use knowledge of statistics, mathematics, and linguistics to analyze and process text materials. According to relevant viewpoints in linguistics, language is an important reflection of cognition. Exploring cognition is a necessary step in analyzing the decision-making process and internal mechanism, while the text content reflects the decision-makers’ cognition and beliefs. Therefore, mining textual data is a necessary prerequisite for measuring constructs in future research on organizational or individual behavior. It can be seen from this that, although text analysis does not occupy a core position in empirical research, but is only a method to explore themes or measure constructs, it is a necessary precursor to studying the relationships between constructs [21]. Text analysis was initially employed predominantly in the information and intelligence domain, it has progressively established itself as a crucial research approach within the social sciences. As a non-intrusive analytic tool, this method assists researchers in impartially and objectively examining facts and formulating conclusions [22]. Nvivo software is extensively utilized as a data analysis software in qualitative research. It offers researchers valuable assistance in collecting, organizing, analyzing, and presenting textual data. By utilizing this software, researchers can enhance the rigor and reliability of their qualitative research [23].

Firstly, this article conducted a segmentation analysis of the policy texts pertaining to the one-vote veto system in the target provinces. A high-frequency thesaurus was formed based on the segmented words, and Nvivo software was utilized to generate a word cloud diagram for each province. This allowed for a macro-level examination of similarities and differences in the policy texts. Secondly, this article used text content and text form as primary coding indicators and further developed more detailed and specific secondary coding indicators. The policy texts involving the one-vote veto in the target provinces were subjected to coding analysis from both the content and form perspectives. This micro-level analysis enabled an examination of annual changes in the policy texts related to the one-vote veto system promulgated by the provinces. Drawing on the aforementioned text analysis methods, a systematic discussion of the evolving characteristics and internal logic of the Chinese government’s utilization of the one-vote veto system is achieved by combining macro and micro perspectives.

### 3.2 Sample selection and data sources

This paper selects Fujian, Hubei, and Gansu as the target provinces of the study. The main reason is that these three are the provinces that have issued the most policy texts involving one-vote veto content among the provinces in eastern, central and western China. Therefore, a comparative analysis of these three provinces is easier to distinguish the evolution characteristics and internal logic of China’s one-vote veto system. In order to facilitate the comparison among the research objects, this paper mainly selects the policy texts of three provinces during the 20 years from 2000 to 2019. Relevant policy texts are mainly obtained from the Peking University legal database website, and the retrieval condition is *full text*: *one-vote veto*. After preliminary processing of the policy texts, only the policy texts issued by the provincial people’s congresses, provincial governments, and provincial government departments were retained, and duplicate texts were eliminated, and a total of 999 target policy texts were obtained. Among them, 412 were from Fujian Province, 361 from Hubei Province, and 226 from Gansu Province. On this basis, extracting the text paragraphs involving one-vote veto content from the original policy text to create a new file, and number the filtered text files according to the rules of *province—time—name* to form the policy text database of this study.

### 3.3 Text encoding

Key information that defines the development and progression of policy texts is frequently embedded within the texts and manifested through fundamental elements. Among them, formal information such as the issuing department and issue number of the policy text determines its effectiveness level, while the text title and subject category describe the substantive content information of the policy text [24]. Therefore, the coding of policy texts not only involves the coding of content features, but also the coding of the formal features of the texts, so as to form a coding index system for quantitative analysis of one-vote veto transmutation features and internal logic.

Drawing on the relevant experience of the existing literature on policy text analysis, and combining the classification methods of policy texts on the Peking University legal database website, this paper sets four secondary coding indicators under the first-level coding indicators of formal features. The first is the release time. So as to describe the change process of relevant policy texts from the time dimension; The second is the text type. Specifically including notices, opinions, and decisions; The third is the issuing department. According to the classification rules provided by the Peking University legal database website, policy texts issued in the name of People’s Congresses are categorized as provincial People’s Congresses. Similarly, policy texts issued in the name of the provincial government as a whole are classified as provincial governments, while policy texts issued in the name of various departments are classified as provincial government departments. As a result, three distinct types of issuing departments are identified: provincial People’s Congresses, provincial governments, and provincial government departments.; The fourth is the level of effectiveness. That is, the vertical level of policy texts in the legal system, specifically including local regulations, local government regulations, local normative documents, local working documents. The selection of the second-level indicators under the first-level coding indicators of content features is mainly based on the setting of local government functions. According to the relevant statements in the *State Council Institutional Reform Plan* issued in March 2018, the Chinese government mainly performs five functions: economic regulation, market supervision, social management, public services, and environmental protection. Based on this, the five items of economic regulation, market supervision, social management, public services, and environmental protection are set as five second-level indicators under the first-level indicators of content features. At the same time, the development planning and evaluation policy texts related to the work of the government departments themselves are uniformly included in the indicators of agency work. Finally, six second-level coding indicators under the first-level coding indicators of content features were constructed. The coding index system is shown in Table 1.

**Table 1:** Coding index system for policy text analysis.

First-Level Indicators	Second-Level Indicators	Text Signature Words
formal features	release time	year, month, day
	text type	notices, opinions, decisions, etc.
	issuing department	Fujian Provincial People’s Congress, Fujian Provincial Government, Fujian Provincial Government Departments, Hubei Provincial People’s Congress, Hubei Provincial Government, Hubei Provincial Government Departments, Gansu Provincial People’s Congress, Gansu Provincial Government, Gansu Provincial Government Departments, etc.
	level of effectiveness	Local regulations, local government regulations, local normative documents, local working documents, etc.
content features	economic regulation	Credit, economic development, tax reform, investment environment, etc.
	market supervision	Production safety, engineering quality, food safety supervision, comprehensive industry regulation, etc.
	social management	Family planning, social security, fire safety work, etc.
	public services	Education, health, personnel training, scientific and technological progress, graduate employment, civilized community, etc.
	environmental protection	Environmental protection, pollution prevention and control, pollutant emission reduction, energy saving and consumption reduction, ecological civilization, etc.
	agency work	Performance appraisal, selection and commendation, democratic appraisal, action planning, work style construction, etc.

## 4. Analysis of research results

### 4.1 Keyword frequency comparison analysis

**Fig 1:**
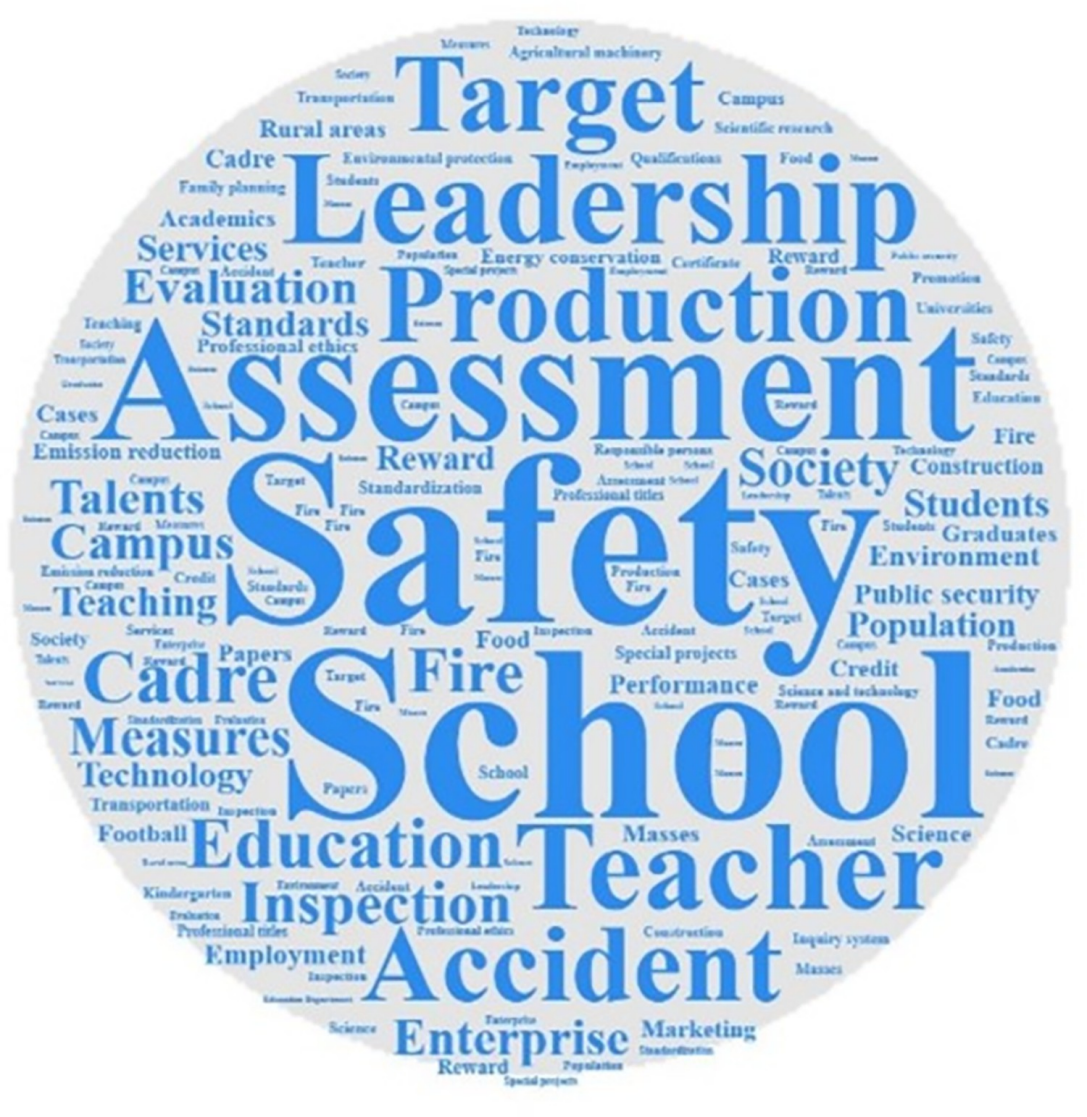
Word cloud map of related policy texts in Fujian Province.

**Fig 2:**
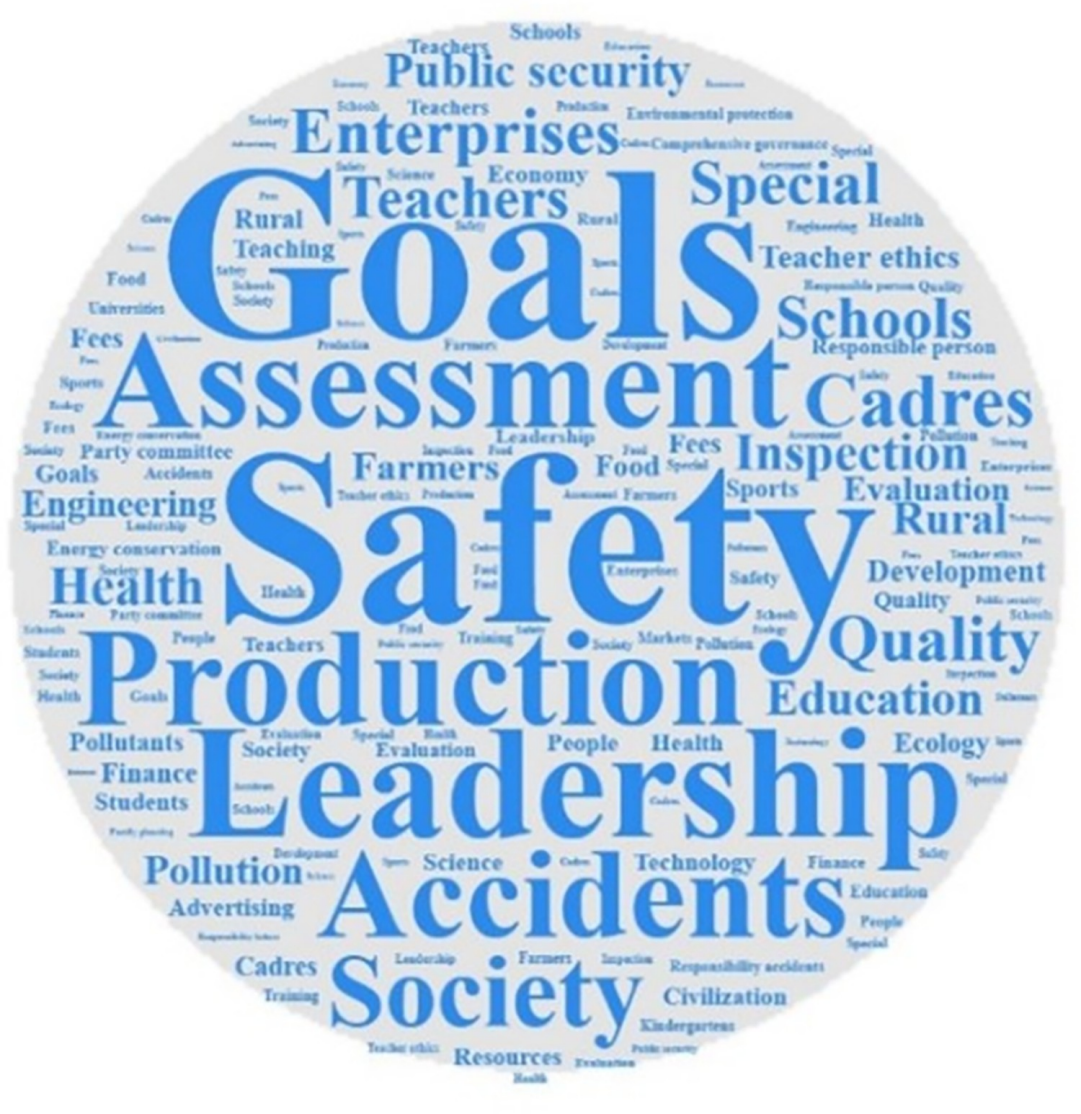
Word cloud map of related policy texts in Hubei Province.

**Fig 3:**
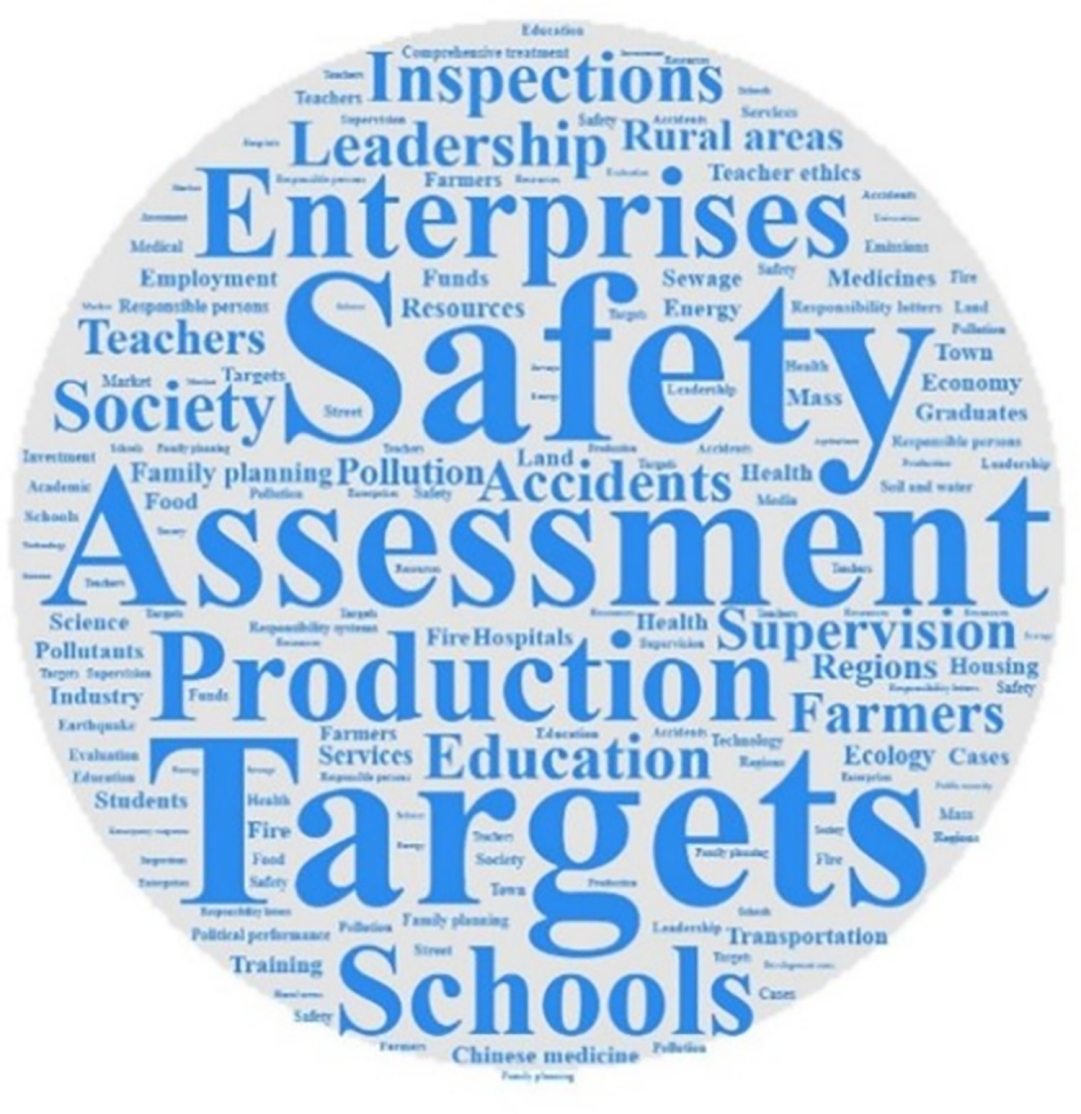
Word cloud map of related policy texts in Gansu Province.

Analyzing the frequency of key words can identify the differences and connections that exist when different local governments use the one-vote veto system. According to different provinces, using ROSTCM6.0 software to conduct word segmentation and word frequency statistics on the policy texts included in the research database of this paper. After removing meaningless and ambiguous keywords, three high-frequency lexicons were formed. Output the top 15 keywords in the frequency of the three provinces to form a high-frequency vocabulary list. At the same time, based on the high-frequency lexicon, use Nvivo software to form a word cloud map of high-frequency words. The word cloud map of the three provinces are shown in Figs 1–3. And the high-frequency vocabulary list of the three provinces are shown in Tables 2–4.

**Table 2:** High-frequency vocabulary list of related policy texts in Fujian Province.

High-frequency Keywords (Frequency)				
Safety (769)	Assessment (473)	Responsibility (466)	School (416)	Production (308)
Leadership (264)	Teacher (259)	Education (254)	Teacher ethics (226)	Accident (196)
Investigation (181)	Target (157)	Society (142)	Evaluation (139)	Inspection (121)

**Table 3:** High-frequency vocabulary list of related policy texts in Hubei Province.

High-frequency Keywords (Frequency)				
Safety (517)	Assessment (512)	Production (326)	Responsibility (297)	Goal (244)
Leadership (221)	Environment (199)	Accident (160)	Enterprise (151)	Environmental Protection (148)
Society (146)	Family Planning (134)	Inspection (109)	Education (106)	Cadres (102)

**Table 4:** High-frequency vocabulary list of related policy texts in Gansu Province.

High-frequency Keywords (Frequency)				
Assessment (381)	Safety (366)	Responsibility (243)	Production (201)	Target (173)
Enterprise (129)	Energy Conservation (125)	Leadership (119)	Environment (117)	Accident (92)
Supervision (91)	School (82)	Inspection (80)	Education (76)	Pollution (75)

Observing the above charts, it can be found that the policy texts issued by the three provinces involving one-vote veto have both common features and certain differences. The similarities are mainly reflected in two aspects. First, vocabulary such as assessment, inspection, and leadership are high-frequency keywords in the three provinces. And the frequency of the word assessment is ranked in the top two. This shows that the one-vote veto system is mainly regarded as an important means of assessing and accountability of leaders in the process of local governments’ application. Although the results of empirical research have shown that the one-vote veto has not exerted its due binding force in the process of evaluating, selecting and appointing leading cadres [25], it is clear that this indicator is still regarded by the provincial government as an important tool for evaluating grassroots officials. Second, safety, production, responsibility, and accidents are all high-frequency keywords in the three provinces. And the word *safety* is the top two keywords in the three provinces. This shows that the one-vote veto system is mostly used by local governments in the field of safety, including production safety and social security, in accountability and accident handling. Such problems are all long-term projects that pay costs immediately and generate benefits in the future. Therefore, adopting the one-vote veto system on such issues can correct the short-sighted bias that local governments have in intertemporal decision-making.

The description of the similarities can identify the commonality of the three provinces in using the one-vote veto system, and the analysis of the differences can help to discover the characteristics of the provinces in using the system. Observing the above charts, it can be found that the differences in the relevant policy texts of the one-vote veto system in the three provinces are mainly reflected in the areas where the system is applied. Among them, words such as *school*, *teacher*, *education*, and *teacher’s morality* are high-frequency keywords in relevant policy texts related to one-vote veto in Fujian Province, which shows that the one-vote veto system in Fujian Province is mainly used in the field of education. As early as 2003, the Education Department of Fujian Province proposed implementing a one-vote veto system for teaching assessment. Since then, this system has been frequently used in educational issues such as school evaluation, teacher morality evaluation, and teacher title evaluation, and has become an important evaluation and supervision mechanism in the education field of Fujian Province. Words such as *environment*, *environmental protection*, *family planning*, and *education* are high-frequency keywords in relevant policy texts of Hubei Province’s one-vote veto. This shows that the one-vote veto system in Hubei Province is more used in the fields of environmental protection, education and family planning. At the same time, the frequent appearance of energy saving, environment, school, education and pollution in Gansu Province’s one-vote veto policy texts shows that Gansu Province’s one-vote veto system is also mainly used in the fields of environmental protection and education.

Based on the observation of the chart, it can be found that although different provinces have distinct local characteristics when using the one-vote veto system, the scope of application of this system basically revolves around education, environmental protection, family planning and other fields. Different from investment promotion and economic development projects that can obtain immediate high returns, the problems in the above fields generally have the characteristics of low returns, high costs, and difficult governance, and are easily ignored by grassroots governments. Therefore, introducing a one-vote veto indicator in the governance of such issues can effectively restrain the behavior of grassroots governments, thereby achieving greater governance effects at a lower cost. It can be seen from this that it seems understandable to implement the one-vote veto system in this type of field, but the inertia of the system itself determines that once a system is fixed in a field, it will cost a lot of money to change it. Taking the issue of one-vote veto in the education field in Fujian Province as an example, from the initial implementation of one-vote veto to teacher assessment, which was only a principled regulation, to the later increasingly refined description of one-vote veto items, this shows that the thinking of one-vote veto has been ingrained in the governance of problems in the field. This situation of abuse will inevitably lead to problems such as rigid incident handling and rigid assessment methods. Obviously, the governance of the one-vote veto indicator is imminent.

### 4.2 Comparative analysis of the semantic network of keywords

**Fig 4:**
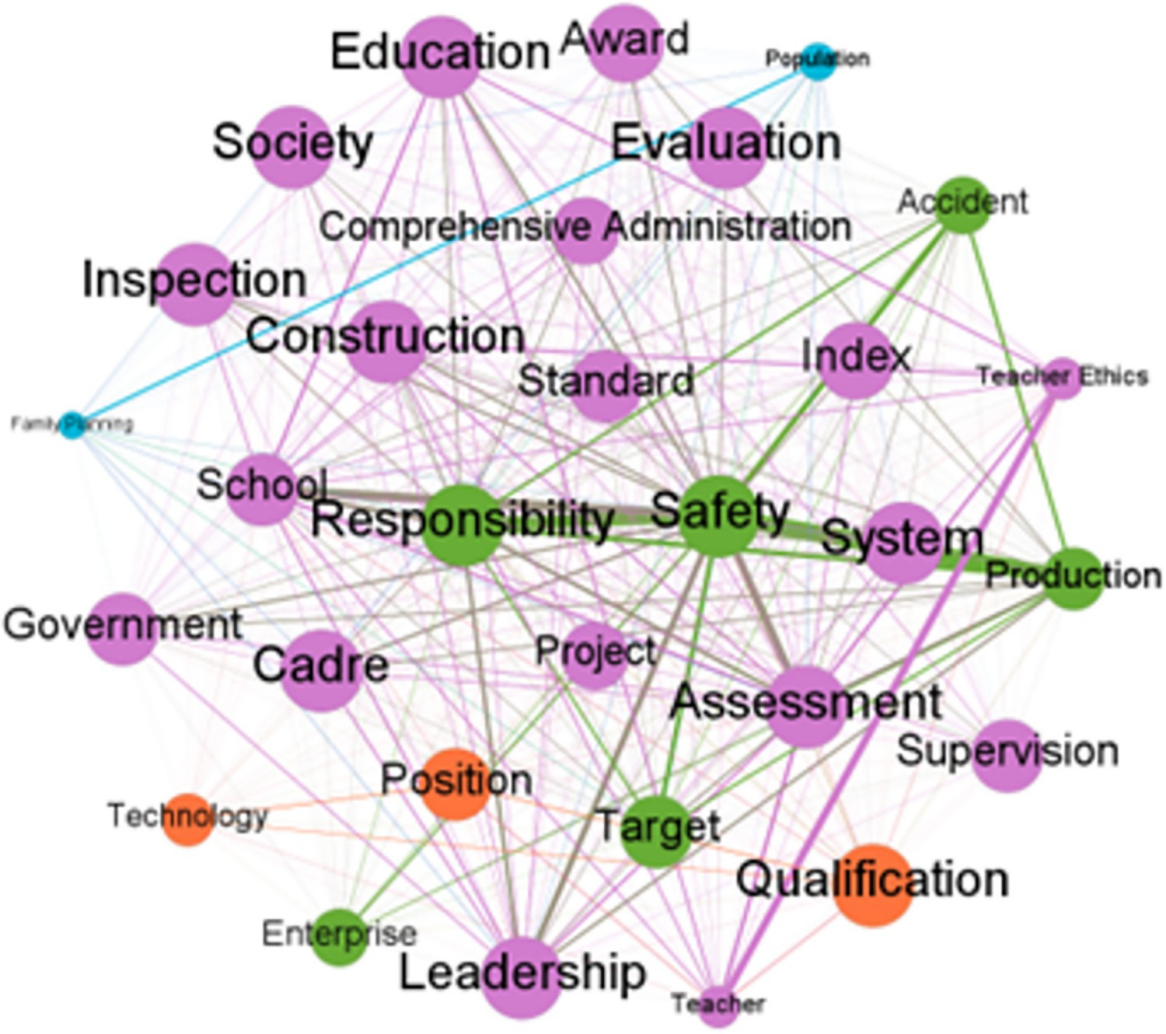
Keyword semantic network of relevant policy texts related to one-vote veto in Fujian Province.

**Fig 5:**
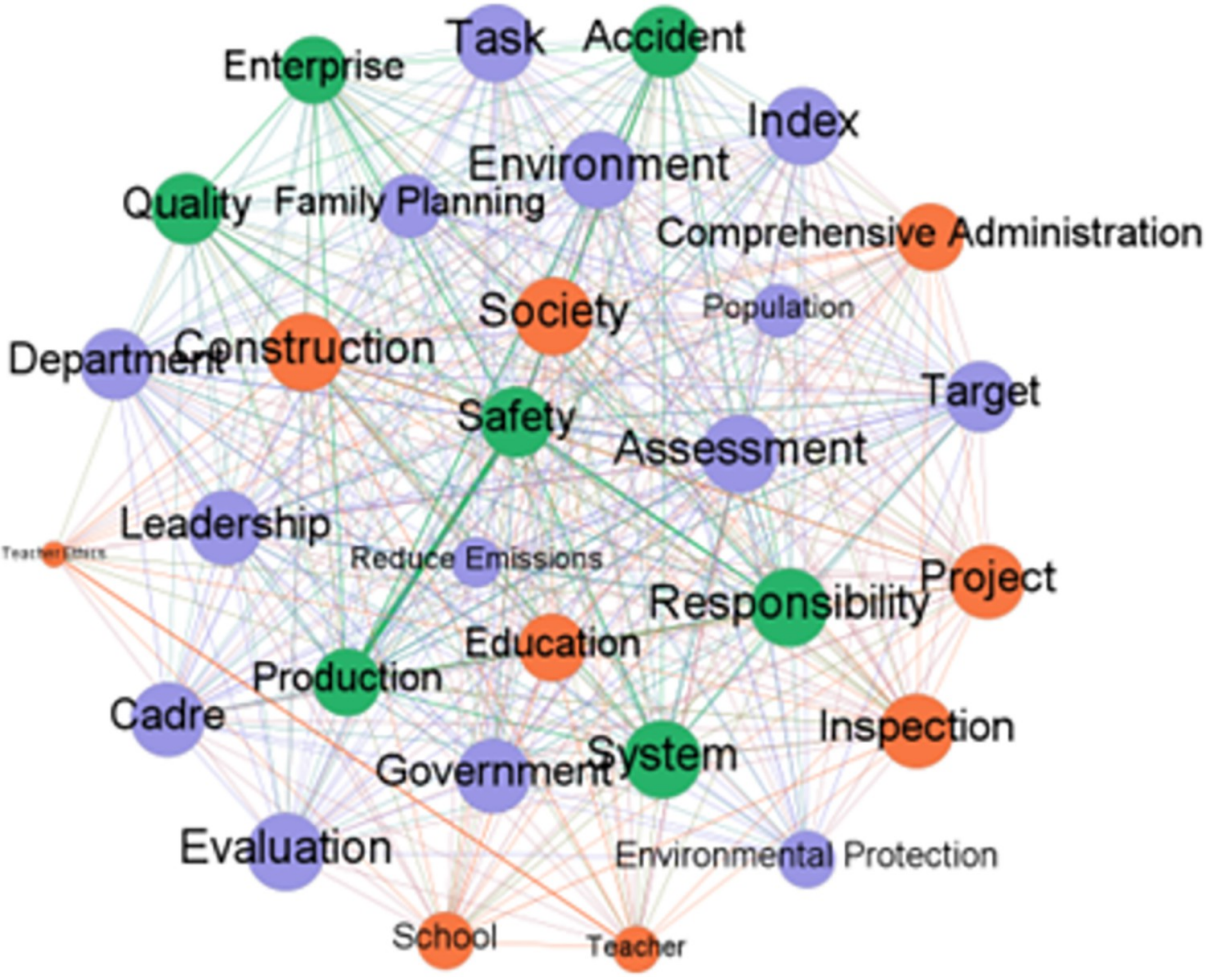
Keyword semantic network of relevant policy texts related to one-vote veto in Hubei Province.

**Fig 6:**
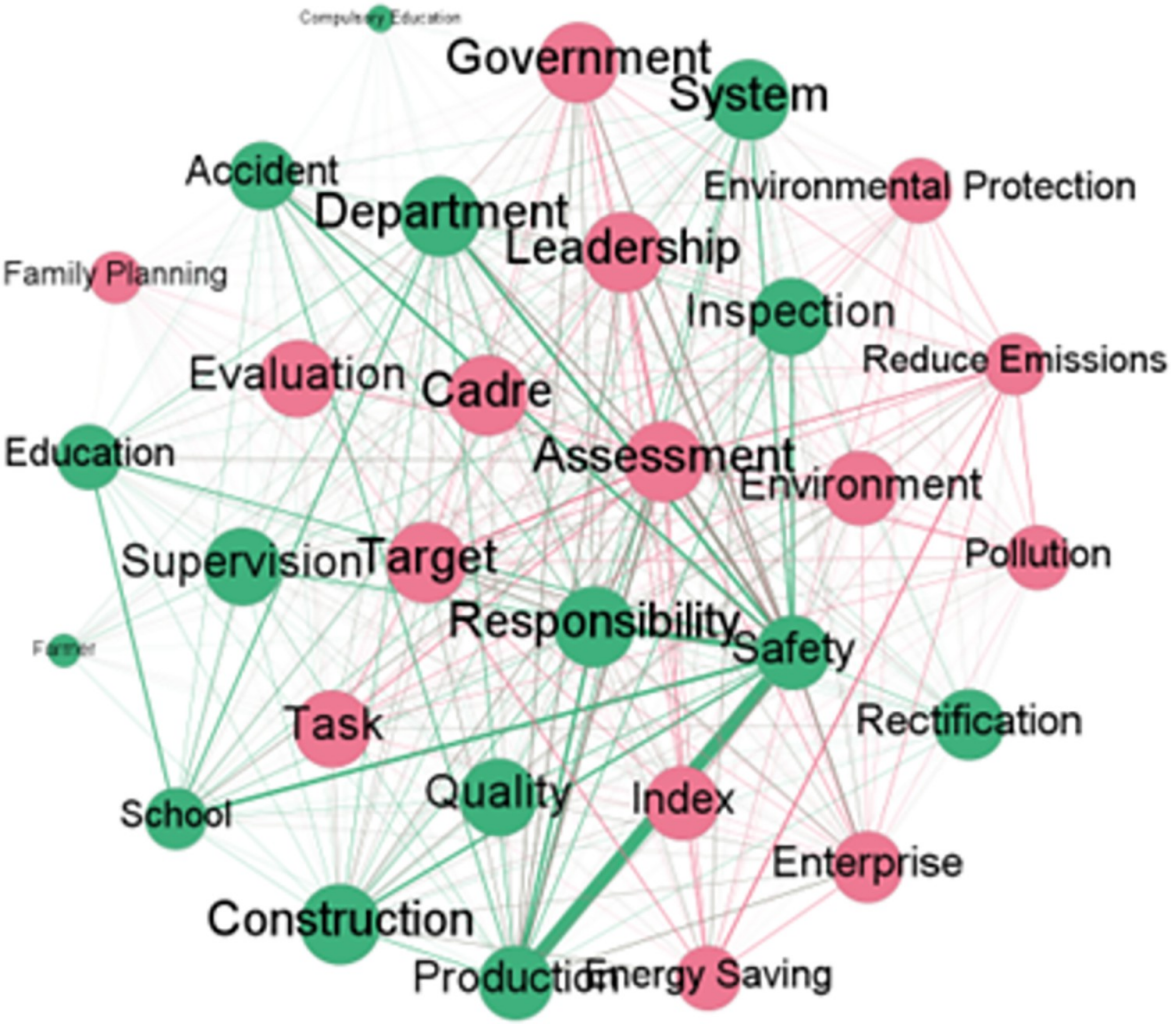
Keyword semantic network of relevant policy texts related to one-vote veto in Gansu Province.

Analyzing the semantic network of keywords can more clearly identify the similarities and differences in the application fields of the one-vote veto system in different local governments, thereby further confirming the existing conclusions. Based on the word frequency statistical data formed above, the top 30 keywords in the three provinces were extracted to construct a keyword matrix, and based on this, a keyword semantic network was formed, as shown in Figs 4–6.

Looking at the above three keyword semantic networks, we can find that there are clear similarities in the application of the one-vote veto mechanism in the three provinces. Specifically, in the three semantic networks, the words *safety*, *production* and *responsibility* are all key words in each network, and they are closely related to each other, forming a clearer secondary network. This shows that the field of production safety is undoubtedly a key area for the application of the one-vote veto mechanism, and it also clearly reflects that the Chinese government attaches great importance to the issue of safety, that is, using a one-vote veto such a strong negative incentive measure to ensure the absolute safety of industrial production activities.

At the same time, the differences in other words related to the three words *safety*, *production* and *responsibility* show that the three provinces have also formed different specific governance strategies when using the veto system to deal with issues in the field of production safety. First, in the keyword semantic network of Fujian Province, the word *target* appears frequently and is closely related to words such as *safety* and *production*. This shows that when dealing with production safety issues, Fujian Province tends to use veto items as a negative assessment target and improve the production safety awareness of grassroots officials through target management. Second, in the keyword semantic network of Hubei Province, the words that are closely related to words such as *safety* and *production* are *system*, *accident*, *quality*, *enterprise*, etc. This shows that, on the one hand, Hubei Province often adopts systematic means when using the one-vote veto system to deal with problems in the field of production safety. On the other hand, enterprise production quality and enterprise accident rate are the key indicators that Hubei Province focuses on when using the one-vote veto to deal with production safety issues. Third, compared with Fujian and Hubei in the eastern and central regions, Gansu Province in the western region has more words associated with the three words of *safety*, *production* and *responsibility* in its keyword semantic network, which undoubtedly shows that Gansu Province requires more refinement when using the one-vote veto system to deal with production safety issues. At the same time, the fact that words such as *supervision*, *inspection*, *rectification*, and *education* occupy a high proportion in the keyword semantic network indicates that, unlike Fujian Province, which adopts the target management method, Gansu Province adopts more process management methods to deal with production safety issues. This undoubtedly reflects from the side that Gansu Province will more frequently adopt the severe negative incentive measure of veto when dealing with production safety issues.

In addition to the similarities and differences in the use of the one-vote veto system among the three provinces in the field of production safety, the more obvious differences among the three provinces in applying the one-vote veto mechanism are mainly reflected in the application fields and the degree of detail of the application of the one-vote veto mechanism in different fields. Specifically, according to the clustering of keywords in the semantic network, it can be found that, first of all, the one-vote veto mechanism in Fujian Province is mainly used in the fields of safe production, education, family planning and science and technology, and the keywords related to the field of education constitute the largest secondary network in the semantic network. This shows that the field of education is undoubtedly the key area for Fujian Province to use the one-vote veto system. At the same time, the appearance of words such as *leadership*, *cadre*, and *award* in the semantic network further shows that Fujian Province is trying to strengthen the construction of teachers’ ethics and style in the province by linking the one-vote veto system with matters such as job promotion and salary rewards. Secondly, the one-vote veto mechanism in Hubei Province is mainly used in the fields of safe production, environmental protection and education. In addition to the field of safe production, it has also formed a relatively obvious secondary network in the fields of environmental protection and education. This shows that Hubei Province has obvious field generalization characteristics in the application of the one-vote veto system, that is, relying on the strong negative incentive measure of the one-vote veto system to reduce the possibility of grassroots officials making mistakes or violating regulations in the governance of issues in various fields. Finally, the keyword semantic network diagram of Gansu Province shows that in addition to safe production, the field of environmental protection constitutes another core field where the province uses the one-vote veto system. Moreover, in the secondary network formed by keywords related to environmental protection, the appearance of words such as *energy saving*, *reduce emissions*, and *pollution* also shows that, similar to Gansu Province strengthening the enforcement of the one-vote veto system in the field of safe production through process management, in the field of environmental protection, the province also ensures the smooth implementation of the one-vote veto system in the field of environmental protection by providing detailed descriptions of specific environmental protection behaviors.

The differences in application fields reflect the careful consideration of different local governments’ conditions in their respective regions when applying the one-vote veto mechanism. Specifically, for Fujian Province, which is located in the developed eastern region, in order to improve its credibility and social cohesion, the local government’s policy programs mostly focus on public service projects that can improve people’s satisfaction. This has led to the application of the one-vote veto mechanism in areas of people’s livelihood such as education and technology. For Gansu Province, which is located in the underdeveloped western region, on the one hand, it has relatively few measures to improve people’s livelihood, which prevents it from focusing too much on public services. On the other hand, the relatively fragile local ecological environment forces the local government to pay attention to environmental protection issues, leading to its one-vote veto mechanism being mainly used in the field of environmental protection. As for Hubei Province, which is located in the central region, it has the characteristics of both the eastern and western regions. On the one hand, its relatively good economic situation enables it to partially meet people’s needs for high-quality public services. On the other hand, environmental protection issues that arise in the process of developing local economies also need to be taken seriously. The combined effect of these two factors has led Hubei Province, located in the central region, to introduce a one-vote veto mechanism in the fields of education and environmental protection.

### 4.3 Comparative analysis of the formal features of the one-vote veto policy text

The formal features of the policy text include the release time, text type, issuing department, and level of effectiveness of the text. The relevant statistical results are shown in the following charts.

**Fig 7:**
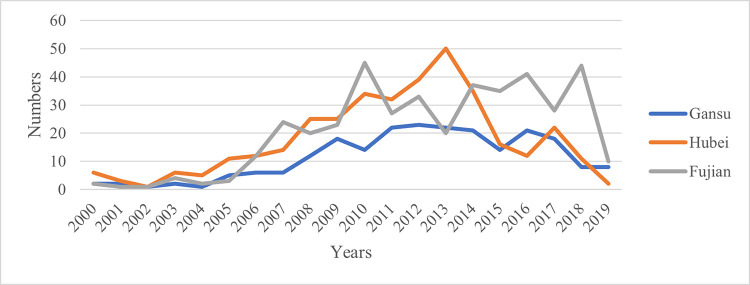
Annual change trend of relevant policy texts in the three provinces.

Fig 7 shows the annual change trend of the relevant policy texts involving one-vote veto content issued by Fujian, Hubei, and Gansu provinces from 2000 to 2019. It can be seen from the figure that although the three provinces are the provinces that publish the most policy texts involving one-vote veto in their respective regions, there are still large differences between the provinces. On the whole, with 2005 as the boundary, the three provinces released relatively few policy texts involving one-vote veto. Since then, the number of relevant policy texts has increased significantly, and reached their respective peaks successively between 2010 and 2013. This means that in the past four years, there has been an upsurge in the use of the one-vote veto system by local governments in China. After 2013, the relevant policy texts of the three provinces have declined to varying degrees, but the overall level is still at a relatively high level. After the *Notice* was issued in 2019, it dropped to a lower level. This shows that the issuance of the *Notice* has a significant corrective effect on the abuse of the one-vote veto system by local governments.

In terms of provinces, the policy texts issued by Fujian Province in the east that involve one-vote veto content are generally maintained at a relatively high level, and the number of policy texts varies greatly between different years. Gansu Province in the west is generally maintained at a relatively low level, and the number of policy texts in different years also has a large range of changes. In Hubei Province, which is located in the central part, the number of policy texts has an obvious trend of first increasing and then decreasing over time. The large annual changes in the number of relevant policy texts in the three provinces indicate that local governments do not rely heavily on the use of this controversial tool. As a mandatory assessment method, the one-vote veto system can effectively urge grassroots government officials to actively perform their duties when used in the work of government centers in a specific period. However, if it is used in general government work, it will easily lead to the behavior of shirking the responsibility of the grassroots government. Therefore, when using this indicator, local governments often adjust its scope of application according to changes in the work of the government center. This is reflected in policy texts that the number of texts often shows large annual changes.

**Fig 8:**
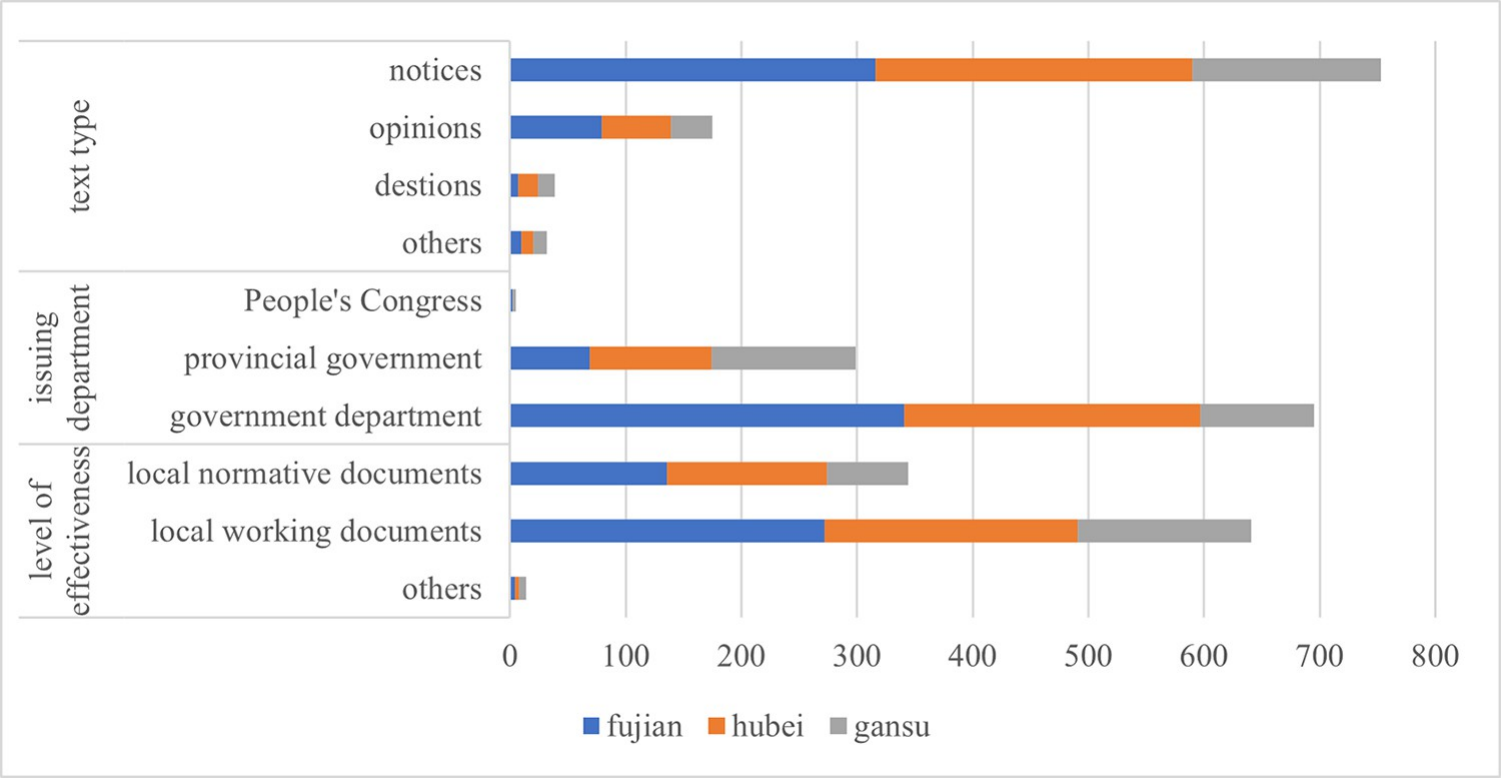
Comparison of formal features of relevant policy texts in the three provinces.

Fig 8 shows the comparison of the formal features of the relevant policy texts involving one-vote veto content issued by Fujian, Hubei, and Gansu provinces.

First of all, in terms of text types, the policy texts issued by the three provinces involving one-vote veto content are mainly notification texts, followed by opinion texts and circular texts. The salient feature of these three types of texts is that they are often the types of texts commonly used when superiors deploy work to subordinates and convey the spirit of important instructions. Policy content generally involves instructions or solutions to specific problems. The resolution of the problem will mean the invalidation of relevant policy texts. Therefore, such policy texts are usually short-term.

Secondly, in terms of the issuing department, the main bodies of the policy texts involving one-vote veto content in Fujian Province and Hubei Province are various government departments, followed by provincial governments. The main issuing department in Gansu Province is the provincial government, followed by various government departments. In addition, very few policy texts are issued by the respective people’s congresses of the three provinces. On the one hand, this characteristic of the main bodies of the policy texts shows that the government still only regards the one-vote veto system as a special tool and applies it to the governance of specific administrative affairs, rather than an overall and comprehensive management method. This will help the government gradually remove the special tool of one-vote veto from the government’s governance toolbox in continuous standardization and legalization of governance tools. On the other hand, the phenomenon that the main bodies of the policy texts are mainly a departmental organization also shows that the one-vote veto indicator has been widely used in the governance of many fields and problems, which is obviously contrary to the connotation of modern governance.

Finally, in terms of level of effectiveness, corresponding to the text type characteristics of relevant policy texts that are mainly notices and opinions, the level of effectiveness of policy texts involving one-vote veto content is mainly local working documents, followed by local normative documents. There are few policy texts that exist at a higher level of effectiveness, such as local regulations and local government regulations. This shows that the policy text involving one-vote veto content is mainly used for the arrangement and communication of specific work between the upper and lower levels within the government.

Based on the above analysis, it can be found that the policy texts issued by the three provinces involving one-vote veto content have distinct common features. The policy texts mainly appear in the form of notices, opinions, and notifications, and the issuing departments are mainly provincial governments and various government departments. The effectiveness level of policy texts are mainly local normative documents and local working documents. The commonality in the formal features of the relevant policy texts of the three provinces shows that when local governments use the one-vote veto system, they mainly regard it as a special means to deal with difficult specific issues. This means that policy texts involving one-vote vetoes should be repealed after the issues are resolved. However, looking at the policy texts issued by the three provinces, it was found that only 57 of the 999 policy texts included in the study were explicitly abolished.

### 4.4 Comparative analysis of the content features of the one-vote veto policy text

**Fig 9:**
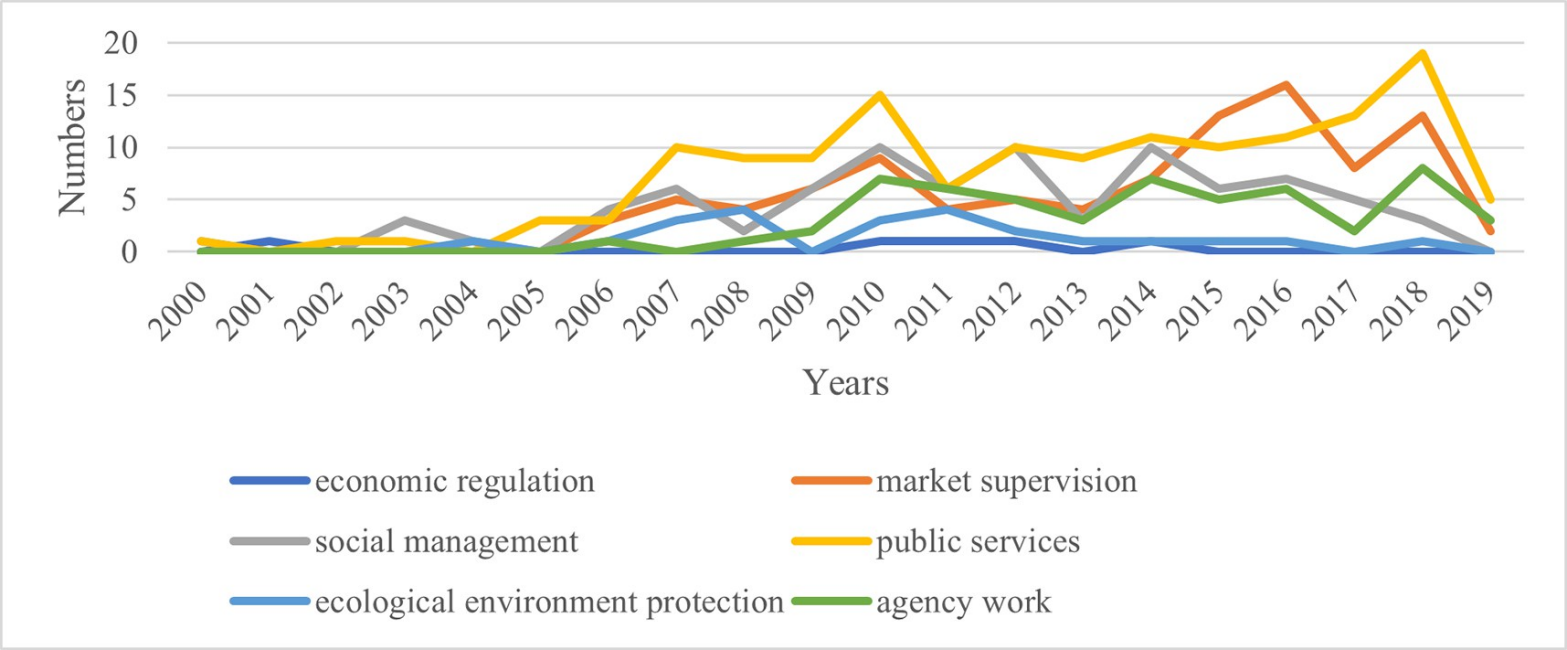
Annual changes in the fields involved in relevant policy texts related to the one-vote veto in Fujian Province.

**Fig 10:**
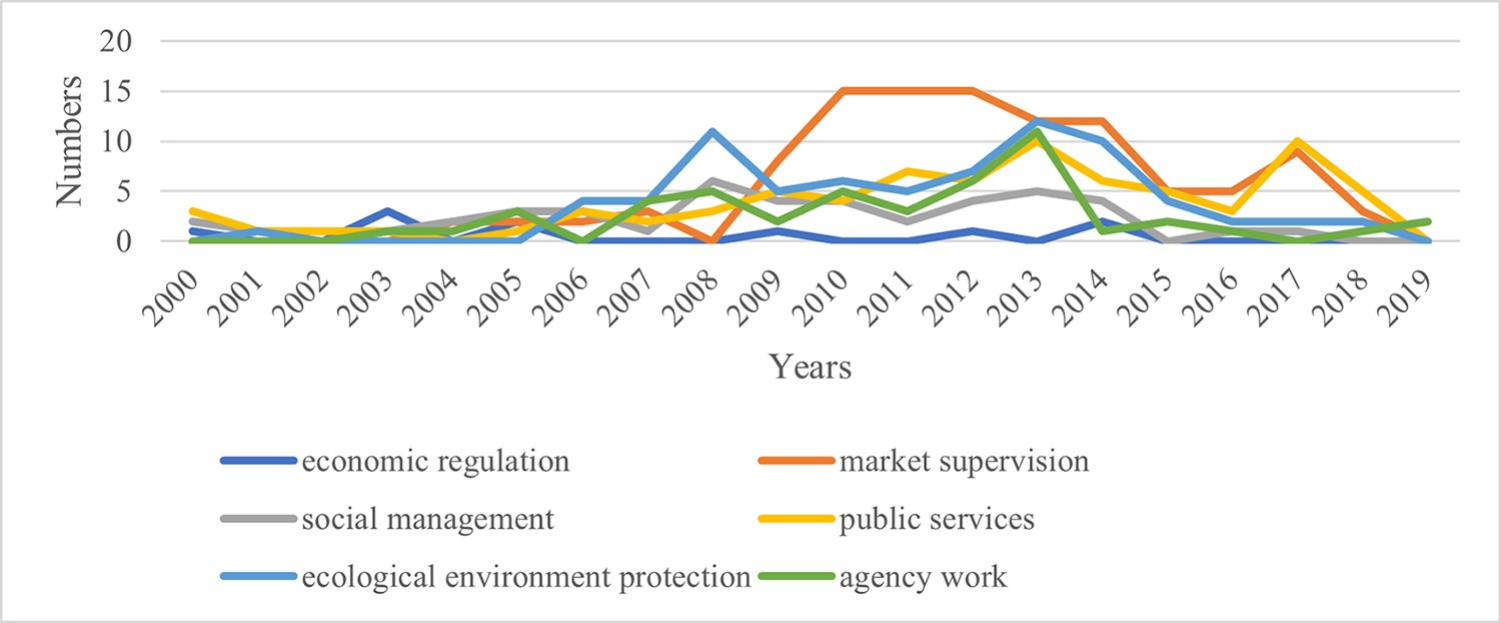
Annual changes in the fields involved in relevant policy texts related to the one-vote veto in Hubei Province.

**Fig 11:**
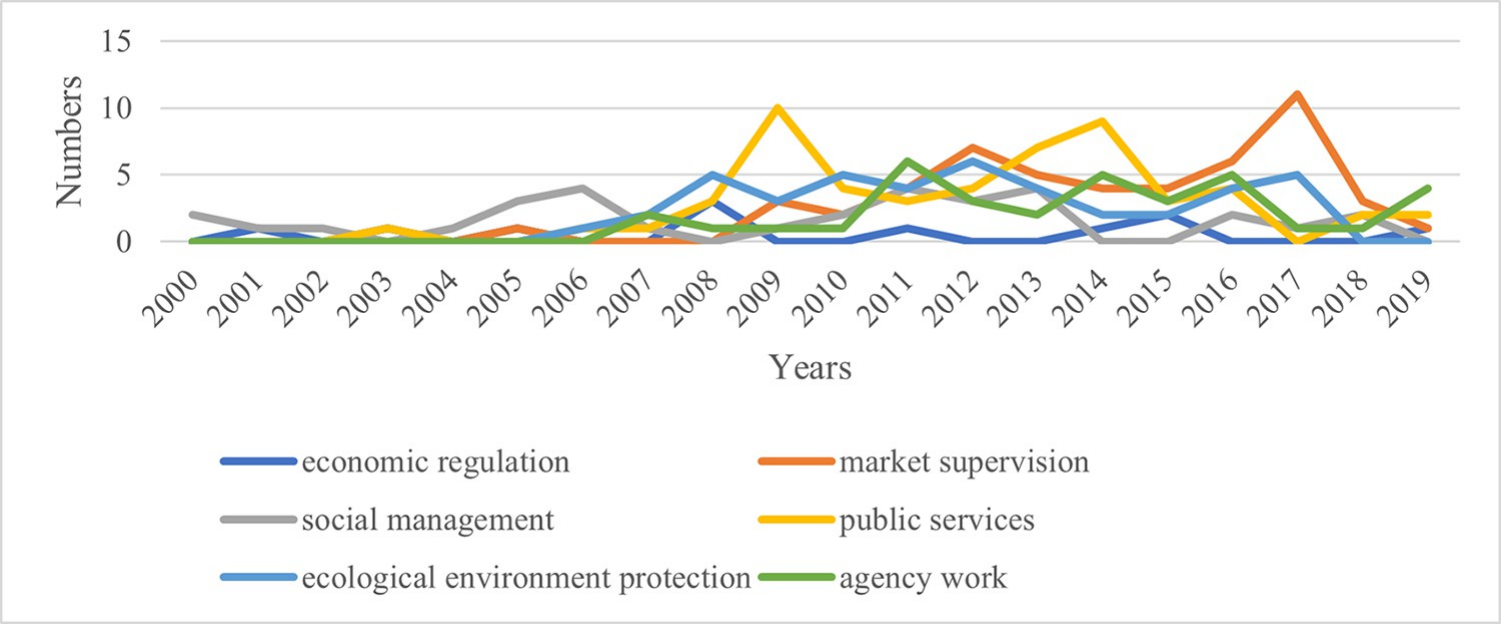
Annual changes in the fields involved in relevant policy texts related to the one-vote veto in Gansu Province.

Analyzing the content features of relevant policy texts related to the one-vote veto can reveal changes in the fields involved in this system and its evolution logic. Using the classification table and matrix coding function of the Nvivo software, based on the year and application field, the annual changes in the fields involved in the one-vote veto related policy texts in Fujian, Hubei, and Gansu provinces are obtained, as shown in Figs 9–11.

As shown in the figure, the projects represented by the curve at the highest point over the years are the main application fields of the one-vote veto system in that province. Observing Fig 9, we can find that before 2004 when the one-vote veto system was not widely used in Fujian Province, it was mainly used in the field of social management. Since then, one-vote veto policy texts in the public service sector have begun to increase. Until 2014, the public service sector was the main application area of the one-vote veto indicator in Fujian Province. At the same time, the number of policy texts related to one-vote veto in the field of market supervision has also increased rapidly, and together with public services, it has become the main application field of the one-vote veto system in Fujian Province in recent years. Observing Fig 10, it can be found that the areas involved in the one-vote veto policy texts dominated by Hubei Province in different years have obvious phases. It has gone through the evolution from the field of social management to the field of environmental protection, and then to the field of market supervision and public services. This change is obviously closely related to the differences in the central work of Hubei Province over the years. Observing Fig 11, it can be found that although Gansu Province has released relatively few policy texts involving one-vote veto content, the dominant field of its application still has a general evolution track from the field of social management, to the field of public services, and then to the field of market supervision.

Based on the above analysis, it can be found that although the three provinces are located in different regions and have different degrees of application of the one-vote veto indicator, there are still some commonalities. On the one hand, it can be seen from the time dimension that the early one-vote veto indicators in the three provinces were mainly used in the field of social management, involving family planning, social security and other matters. Since then, it has been mainly used in the fields of environmental protection, market supervision and public services, involving pollution control, production safety, education and medical care. Although the fields of the problems are different, these problems are often social issues that are closely related to the people and cause great difficulty with governance. If such problems cannot be properly handled, it will cause great damage to the credibility of the government. Therefore, provincial governments often take special measures when asking grassroots governments to deal with such problems, that is, to use one-vote veto indicators to ensure the work quality of the governments. On the other hand, observing the above charts, it can be found that although the three provinces have used the one-vote veto system in the six tasks, the frequency of using this tool in economic regulation is significantly lower than in other fields. This shows that when the provincial government uses the one-vote veto indicator, it is mainly regarded as a special means to regulate and allocate the behavior and attention of the grassroots government. This is mainly manifested in areas where grassroots governments pay less attention to market regulation, public services, and environmental protection. Provincial governments have increased the importance of grassroots governments by introducing one-vote veto indicators. For the governance of economic issues that can bring immediate high returns, since the grassroots government has invested more energy, there is no need to increase its attention in the form of a one-vote veto.

## 5. Conclusion and discussion

This paper uses Nvivo software to conduct text analysis on the policy texts involving one-vote veto content in Fujian, Hubei, and Gansu provinces from 2000 to 2019. The evolution characteristics and internal logic of China’s one-vote veto system, and draw the following conclusions.

First of all, the relevant expressions of one-vote veto have been continuously refined. In Fujian, Hubei, and Gansu provinces’ early policy texts involving one-vote veto content, they often only stipulated in principle the matters of one-vote veto, and did not involve specific content requirements. Take Fujian Province as an example. Before 2005, Fujian Province proposed to implement a one-vote veto system for family planning, comprehensive management of social security and environmental protection. The relevant description in the policy text only stipulates the authority for exercising this power, but does not specify the circumstances of a one-vote veto. This principled expression can not only exert the specific restraint effect of the one-vote veto system on the grassroots government, but also prevent the behavior of the government from being overly restrained. This can ensure a certain degree of flexibility in the action of the grassroots government. However, by generalizing the application field of this system, the description of one-vote veto items in the policy text has also been continuously refined. In a notice issued by Fujian Province in 2016 on the assessment and evaluation of characteristic schools for youth campus football, there were as many as ten descriptions involving the one-vote veto system. And whether to offer football classes and whether to participate in football games in the schools in the jurisdiction are written into the policy text as a clear one-vote veto indicator. Although the refinement of the description can facilitate the development of the work of the grassroots government, at the moment when the one-vote veto index pervades all fields, if all specific tasks are assessed in the form of one-vote veto, it will definitely cause huge work pressure on the government. In addition, the complexity and refinement of the description will bring the risk of involution of the one-vote veto system. Failure to standardize and innovate this special means of governance in time will inevitably lead to a dilemma in the process of modernizing national governance.

Secondly, the exit mechanism for one-vote veto matters is gradually weakening. Based on the comparative analysis of the formal features of the policy texts related to one-vote veto in the three provinces, it is found that policy texts involving one-vote veto content are generally issued by provincial governments and various departments. And its types are usually local regulatory documents or local working documents such as notices and opinions. Its lower level of effectiveness means that relevant policy texts should be abolished in time after the issues involved are resolved. However, after analyzing the policy texts of the three provinces, it is found that among the many policy texts released every year involving one-vote veto content, only a small number of them have been explicitly abolished. And the abolished policy texts are mostly concentrated before 2016. For example, in the four years from 2016 to 2019, the three provinces issued a total of 225 policy texts involving one-vote veto content. However, as of the end of 2019, none of them has been explicitly abolished, and some of the policy texts have clearly failed to meet the needs of social development. As an important basis for government governance, if outdated policies cannot be abolished in time, it will not only increase the work pressure of grassroots governments, but also inevitably cause conflicts between different policies with the accumulation of policy texts, which will damage the prestige of the legal system.

Finally, the field of application of the one-vote veto is becoming more and more generalized. Based on the comparative analysis of the content features of the policy texts related to one-vote veto in the three provinces, it is found that the application field of the one-vote veto system was relatively single before 2005, mainly concentrated in the field of social management. After 2005, the number of policy texts involving one-vote veto content began to increase sharply, and its application fields became more and more generalized. When solving problems in the fields of market management, public services, and environmental protection, provincial governments have gradually regarded the one-vote veto as an important tool to restrain the behavior of grassroots governments. As an assessment method with a strong deterrent effect, introducing the one-vote veto into the governance of a certain issue can indeed significantly increase the importance of grassroots governments and enable the will of provincial governments to be implemented. However, if the one-vote veto system is used extensively in the assessment of many fields, it will cause the grassroots government to struggle to deal with it, and then resort to fraud. This will undoubtedly damage the rigid incentive effect of the one-vote veto system and the credibility of the government.

This article discusses the evolution characteristics and internal logic of the one-vote veto system implemented in China in the past two decades. Upon analyzing policy texts, it becomes evident that the Chinese government demonstrates a propensity to exploit the one-vote veto system. While it is undeniable that this strict evaluation measure has its advantages in promoting officials’ accountability, it also has the potential to encourage detrimental behaviors such as indifferent compliance and evading responsibility through collaboration at the local level. Consequently, the discussion surrounding the one-vote veto issue brings forth a fresh perspective on the widespread phenomenon of local government officials shirking their obligations.

For the purpose of simplifying the research, this article only uses the policy texts issued by Fujian, Hubei, and Gansu provinces involving one-vote veto content as the research data source. Nevertheless, as the three provinces in the eastern, central and western regions that have issued the most policy texts involving one-vote veto content, an analysis of their policy texts can still provide a more comprehensive picture of the evolution characteristics and internal logic of China’s one-vote veto system. Future research will build upon this as a foundation. Firstly, the research scope will be expanded to include additional provinces in order to corroborate the validity of the study’s findings through a larger sample size. Secondly, utilizing an institutional behavior analysis perspective, the correlation between the veto system and evasive behaviors regarding responsibility will be investigated, aiming to deepen the exploration of pertinent matters in the realm of responsible politics within China.

## Supporting information

S1 Dataset The data set used in this article for discussion and analysis. (ZIP)
