# Supplementary figures and images for: The evolutionary features and internal logic of the one-vote veto system: A Nvivo analysis based on the policy texts of the three provinces in the east, middle and west
Source: PLoS One. 2024 Jul 5;19(7):e0306535. doi: 10.1371/journal.pone.0306535 (PMC11226072; doi:10.1371/journal.pone.0306535)

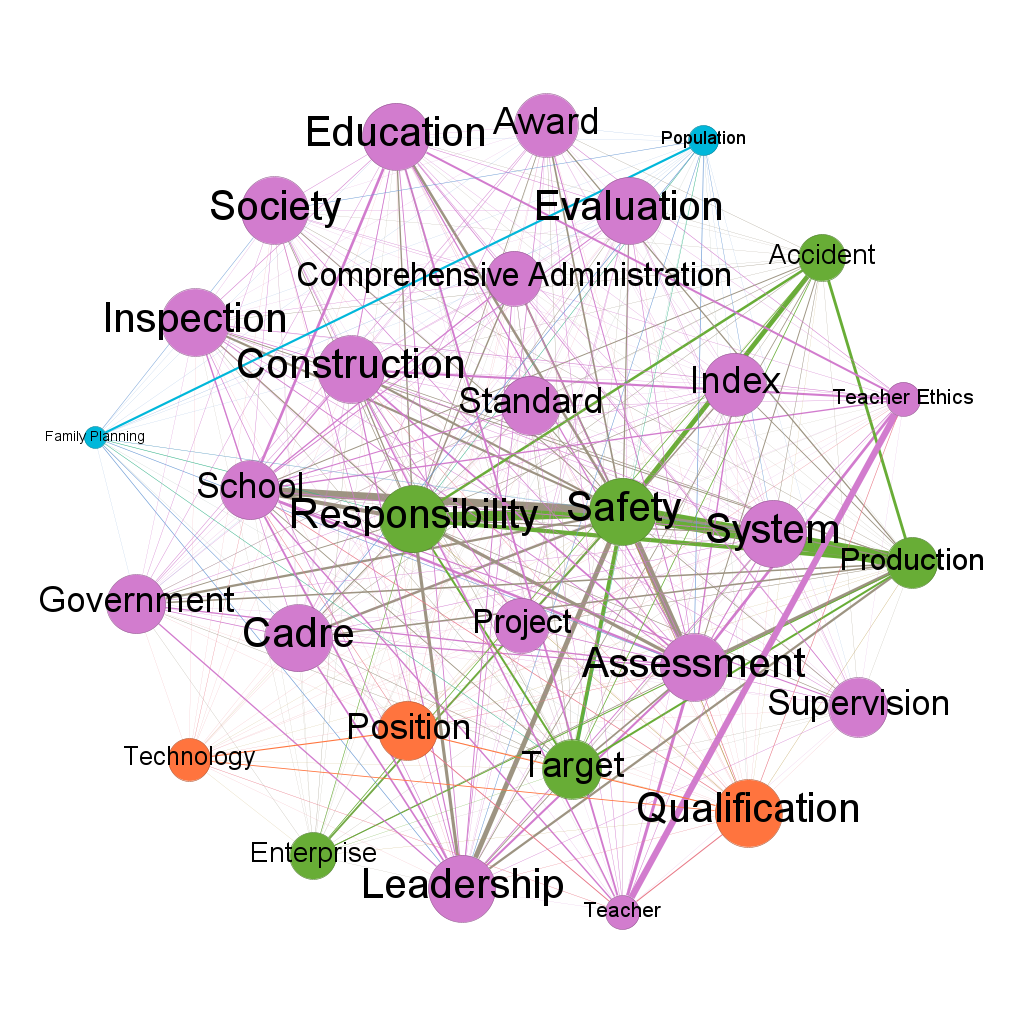

Supplement: S1 Dataset — (ZIP) [file pone.0306535.s001.zip › S1 dataset/Keyword semantic network of Fujian Province.png]

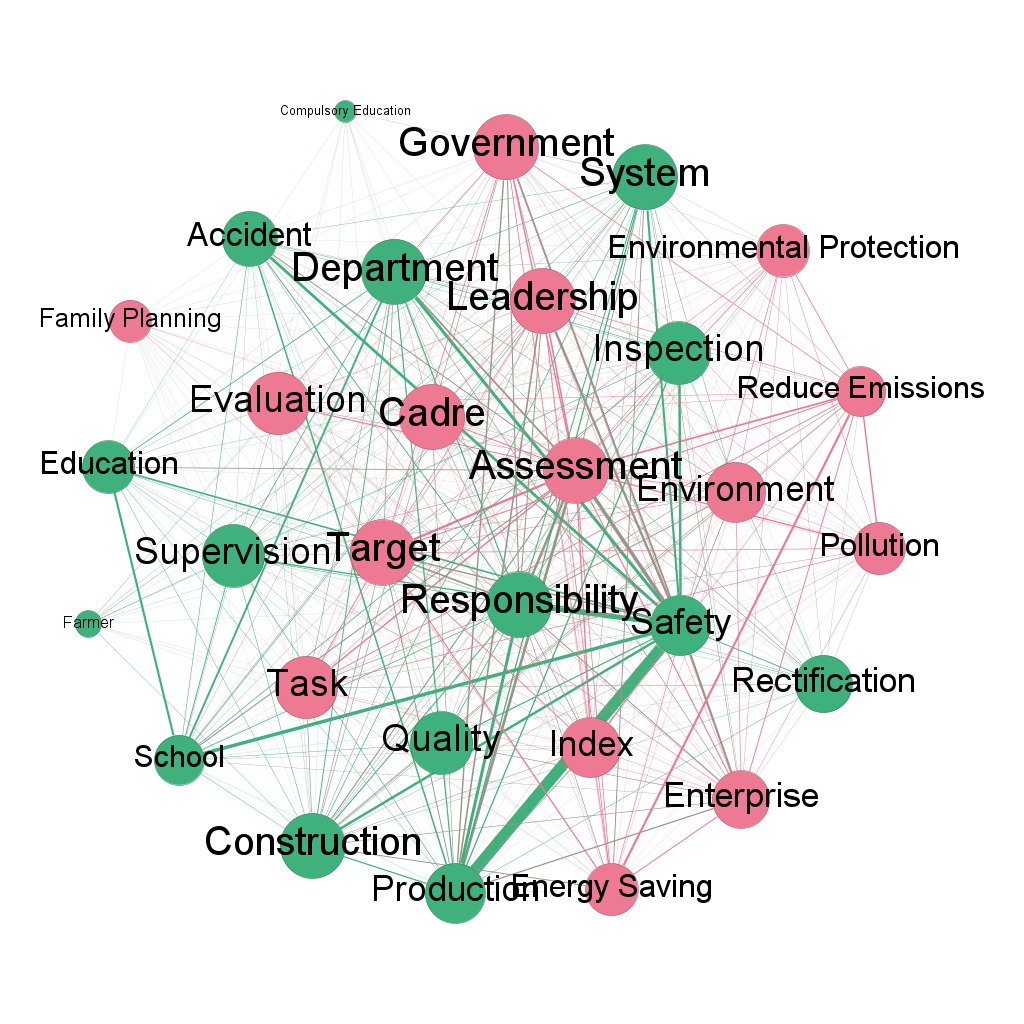

Supplement: S1 Dataset — (ZIP) [file pone.0306535.s001.zip › S1 dataset/Keyword semantic network of Gansu Province.png]

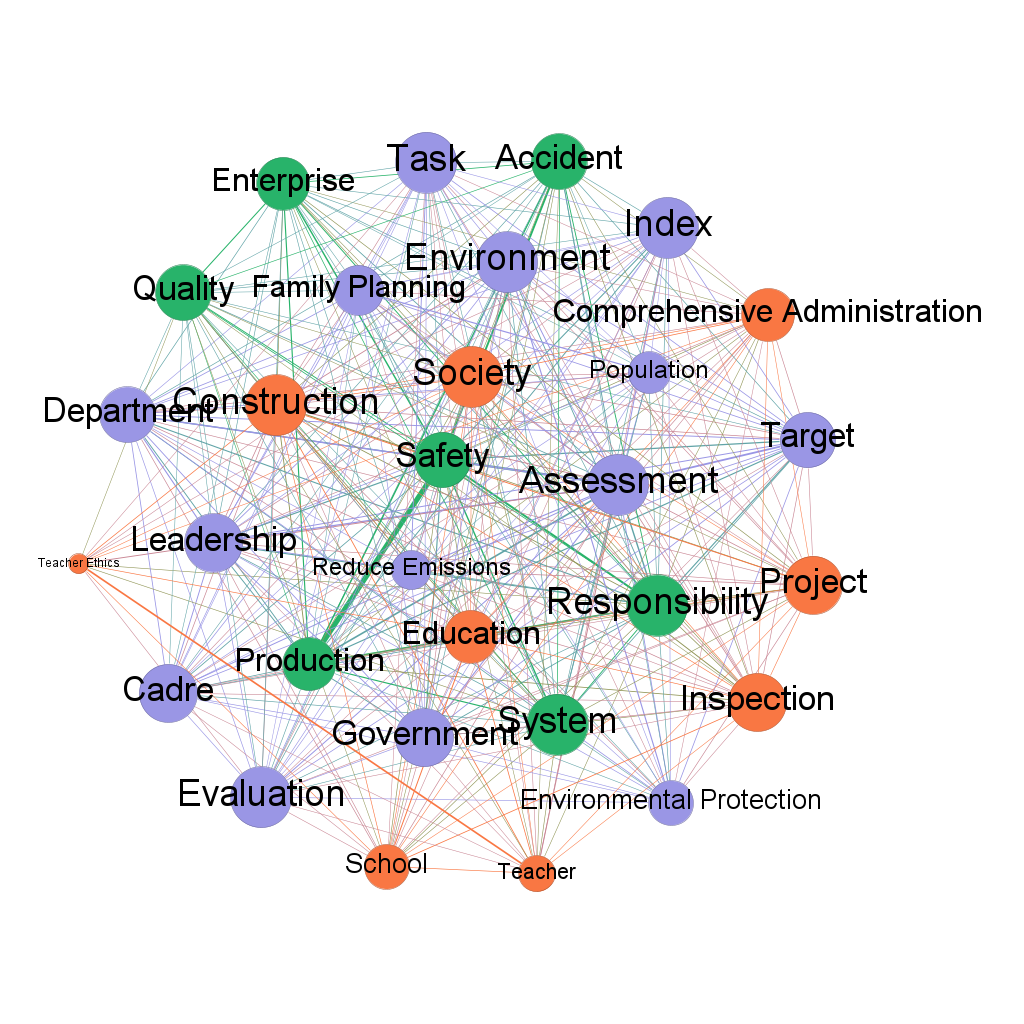

Supplement: S1 Dataset — (ZIP) [file pone.0306535.s001.zip › S1 dataset/Keyword semantic network of Hubei Province.png]

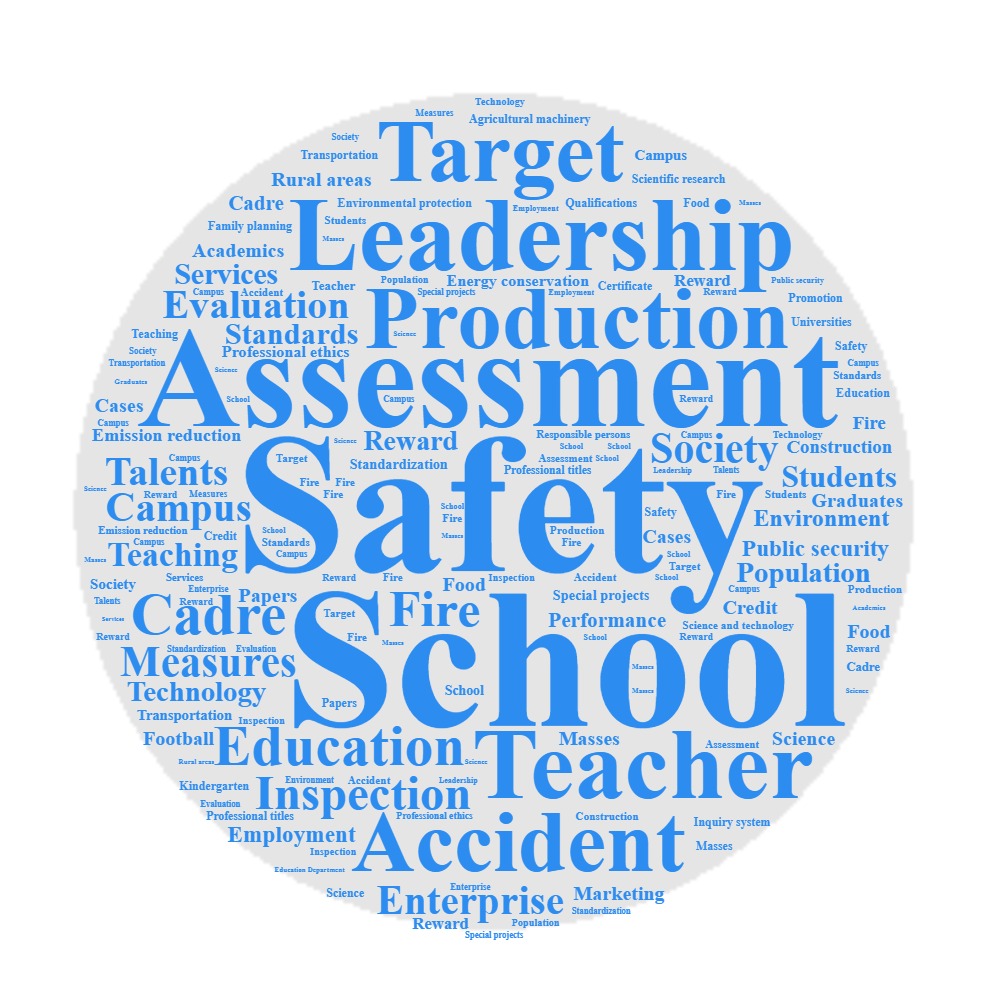

Supplement: S1 Dataset — (ZIP) [file pone.0306535.s001.zip › S1 dataset/Word Cloud Map of Fujian Province.jpg]

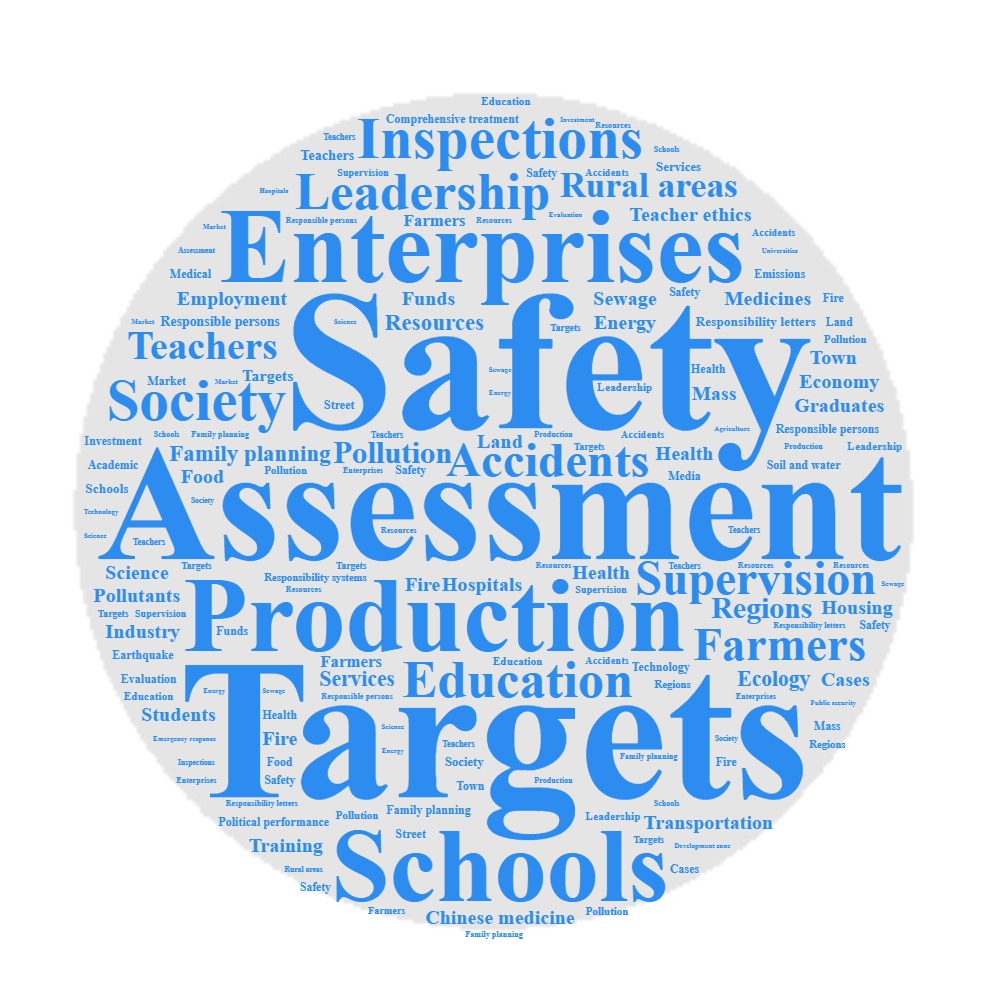

Supplement: S1 Dataset — (ZIP) [file pone.0306535.s001.zip › S1 dataset/Word Cloud Map of Gansu Province.jpg]

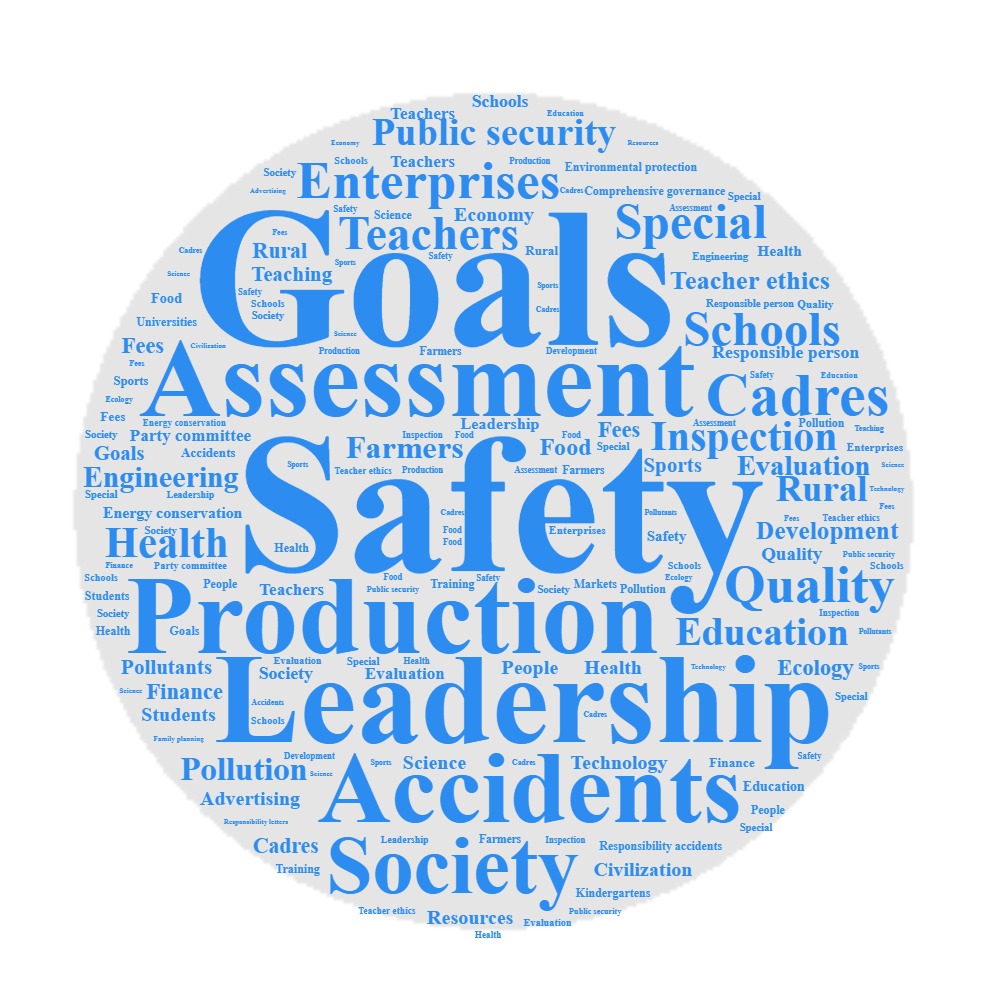

Supplement: S1 Dataset — (ZIP) [file pone.0306535.s001.zip › S1 dataset/Word Cloud Map of Hubei Province.jpg]
